# Phylogenetic Analysis of the Plant U2 snRNP Auxiliary Factor Large Subunit A Gene Family in Response to Developmental Cues and Environmental Stimuli

**DOI:** 10.3389/fpls.2021.739671

**Published:** 2021-11-17

**Authors:** Shuai Lu, Cong Gao, Yongzhou Wang, Yingying He, Junrong Du, Moxian Chen, Hua Zhao, Hui Fang, Baohua Wang, Yunying Cao

**Affiliations:** ^1^School of Life Sciences, Nantong University, Nantong, China; ^2^CAS Key Laboratory of Quantitative Engineering Biology, Shenzhen Institute of Synthetic Biology, Shenzhen Institutes of Advanced Technology, Chinese Academy of Sciences, Shenzhen, China

**Keywords:** splicing, U2AF65A, stress, gene expression, protein interaction

## Abstract

In all organisms, splicing occurs through the formation of spliceosome complexes, and splicing auxiliary factors are essential during splicing. U2AF65 is a crucial splicing cofactor, and the two typical RNA-recognition motifs at its center recognize and bind the polypyrimidine sequence located between the intron branch site and the 3′-splice site. U2AF65A is a member of the U2AF65 gene family, with pivotal roles in diseases in mammals, specifically humans; however, few studies have investigated plant U2AF65A, and its specific functions are poorly understood. Therefore, in the present study, we systematically identified U2AF65A in plant species from algae to angiosperms. Based on 113 putative U2AF65A sequences from 33 plant species, phylogenetic analyses were performed, followed by basic bioinformatics, including the comparisons of gene structure, protein domains, promoter motifs, and gene expression levels. In addition, using rice as the model crop, we demonstrated that the OsU2AF65A protein is localized to the nucleus and cytoplasm, and it is involved in responses to various stresses, such as drought, high salinity, low temperature, and heavy metal exposure (e.g., cadmium). Using *Arabidopsis thaliana* and rice mutants, we demonstrated that *U2AF65A* is involved in the accumulation of plant biomass, growth of hypocotyl upon thermal stimulation, and reduction of tolerance of high temperature stress. These findings offer an overview of the U2AF65 gene family and its stress response functions, serving as the reference for further comprehensive functional studies of the essential specific splicing cofactor U2AF65A in the plant kingdom.

## Introduction

Transcripts produced by transcription in the nucleus of eukaryotes are not directly translated into proteins. These original transcripts that have been transcribed without modification are called pre-mRNAs. It forms mature mRNA after capping, splicing, and tailing in the nucleus. The splicing of pre-mRNA is completed by a large, dynamic complex called the spliceosome. There are two types of spliceosome, i.e., major and minor spliceosomes (Wahl et al., 2009). Major spliceosomes include U1, U2, U4, U5, and U6 snRNPs (Kaida et al., 2010), and minor spliceosomes include U5, U11, U12, U4 atac, and U6 atac (König et al., 2007; Tidow et al., 2009). The spliceosome is a multisubunit RNA-protein complex. Splicing is also regulated by the *trans*-acting factor-spliceosome, which includes five small nuclear RNAs (U1, U2, U4, U5, and U6) and more than 100 core proteins (Galej, 2018), one of which is U2AF. U2AF is a necessary cofactor for U2snRNP to bind to pre-mRNA branch sites. U2AF consists of two subunits, U2AF65 and U2AF35, and their relative molecular weights are 65 and 35 kDa, respectively.

Among them, the large subunit U2AF65 recognizes the polypyrimidine tract (Py tract) shared among pre-mRNA introns and is mainly composed of uracil. U2AF65 was originally isolated, cloned and expressed in 1992 (Zamore et al., 1992). Studies showed that it contained two functional domains, namely, the RNA binding domain (RBD) and the arginine/serine-rich domain, both of which are necessary for pre-mRNA splicing (RS domain) (Galej, 2018). U2AF65 contains 3 RBDs, which are also known as RNA recognition motifs (RRMs). Among them, RRM1 and RRM2 in the center are responsible for identifying the Py tract in the pre-mRNA, and the third RRM at the C end has special structural characteristics and is mainly involved in the interaction between proteins. This special RRM, also known as the U2AF homology motif (UHM), exists in many splicing factors (Kielkopf, 2004), and it consists of 80 to 90 amino acid residues that form 4 antiparallel β sheets and 2 α helices, presenting a split αβ (βαββαβ) topology (Lunde et al., 2007). The RNA binding activity of the UHM domain is very weak or even absent. The RS domain is generally considered to be involved in the interaction between proteins in splicing bodies and partly mediated by phosphorylation of serine (Fu et al., 1995). The RS domain at the N-terminus of U2AF65 is special and can bind to RNA, but it does not mediate the interaction between protein and protein. Experimental results show that U2AF65 cross-connects with branch sites, which makes it easier for snRNA in U2 snRNP to interact with branch sites (Valcárcel et al., 1996).

The assembly of the spliceosome begins when U1 snRNP binds to GU at the 5′ splice site. The RRM of U2AF35 recognizes the AG at the 3′ splice site and binds to the N-terminal proline-rich region of U2AF65. The two typical RRMs in the center of U2AF65 recognize and bind to the polypyrimidine sequence located between the intron branch site and the 3′ splice site. The C-terminal UHM and splicing factor 1 (SF1) interact to enhance the abovementioned binding. Then, U2 snRNP replaces SF1 and binds to the branch site to form presplicing complex A. The combination of U2 snRNP enables smooth splicing of pre-mRNA (Berglund et al., 1998; Selenko et al., 2003; Kent et al., 2005). The stable binding of U2 snRNP to the branch site requires the RS domain at the N end of U2AF65 and RNP helicases such as UAP65 (Fleckner et al., 1997). Finally, the combination of U5/U4/U6 snRNP completes the assembly of the spliceosome. After two consecutive transesterification reactions, intron removal and exon connection are completed. The binding of U2AF65/U2AF35 is quite stable and released from pre-mRNA before the activated spliceosome catalyzes the splicing reaction. The binding to branch site U2AF65 is relatively weak. U2AF65 also plays an important role in alternative splicing. The alternative splicing of an exon may result in a weaker binding force with U2AF65 due to the interference of the polypyrimidine sequence of the adjacent intron by purines. Serine/arginine-rich splicing activates proteins, strengthening the aforementioned weak binding (Graveley et al., 2001). U2AF is involved in apoptotic pathways. As a prototype of death receptors, Fas has been implicated in non-apoptotic signaling pathways (Guégan et al., 2019). At the early stages of apoptosis, U2AF65 is cleaved by cysteine proteases, which affects the alternative splicing of Fas (Izquierdo, 2008). The U2AF–RNA interaction is weakened in hypoxic cells, resulting in the inhibition of Fas-mediated apoptotic pathways and, ultimately, the promotion of apoptosis (Vilys et al., 2021).

Previous research on U2AF65 mainly focused on animals. U2AF65 is scattered in the nucleus in a stable state (Gama-Carvalho et al., 1997). Further studies indicated that U2AF65 and U2AF35 continuously shuttle between the nucleus and the cytoplasm, which raises the possibility that U2AF may be transported to the cytoplasm together with mRNA (Gama-Carvalho et al., 2001). Subsequent studies reported that U2AF was involved in mRNA transport in both mammals and *Drosophila* (Zolotukhin et al., 2002; Blanchette et al., 2004). Therefore, research has shown that U2AF65 not only binds to the intron polypyrimidine sequence of pre-mRNA in the first step of spliceosome assembly but also binds to spliced modified mRNA. The proteins encoded by these mRNAs are mainly related to cell cycle processes. The transcriptional regulation of U2AF65 and the stable maintenance of chromatin are related (Gama-Carvalho et al., 2006). Minichromosome loss mutants mistakenly lead to the death of fissile yeast because aneuploidy and replication abnormalities may be related to U2AF65 (Takahashi et al., 1994). *In vitro* protein-RNA interaction studies of pre-mRNAs containing either a constitutive or regulated splicing enhancer revealed that U2AF35 directly mediated the interactions between U2AF65 and proteins binding to the enhancers. U2AF35 functions as a bridge between U2AF65 and the enhancer complex to recruit U2AF65 to the adjacent intron (Zuo and Maniatis, 1996). U2AF35-related protein (Urp) is associated with the U2AF65/U2AF35 heterodimer and specifically interacts with U2AF65 through a U2AF35-homologous region and with SR proteins (a large family of RS domain-containing proteins) by its RS domain. Therefore, Urp and U2AF35 may independently position RS domain-containing factors within spliceosomes (Tronchère et al., 1997). Related studies have shown that U2AF65 recognizes the negative regulatory element of human papillomavirus late mRNA (Dietrich-Goetz et al., 1997). U2AF65 is related to the regulation of human papillomavirus or the binding of human immunodeficiency virus (Cumming et al., 2002; Domsic et al., 2003). The splicing factor U2AF65 is functionally conserved in the thermotolerant deep-sea worm *Alvinella pompejana* (Henscheid et al., 2005). The energetic importance of conserved residues for polypyrimidine (Py) tract binding was established by analyzing site-directed mutant U2AF (65) proteins using surface plasmon resonance, which revealed the structural basis underlying the recognition of polypyrimidine tracts by U2AF65 (Sickmier et al., 2006). The components of U2 snRNA and U2AF (65) are related to exonic splicing enhancers (ESEs) in the *Xenopus* oocyte nucleus (Masuyama et al., 2007). Studies have shown that U2AF65 is related to polyglutamine (polyQ) diseases (Tsoi et al., 2011). Increased U2AF65 expression is associated with the total and truncated expression of beta-catenin in high-stage colorectal tumors (Nelson et al., 2012). U2AF65 interacts with TRF1 in humans and *in vitro* and can stabilize the TRF1 protein by inhibiting its ubiquitin-dependent proteolysis. U2AF65 represents a new method of regulating the function of the telomere TRF1 in the human body (Kim and Chung, 2014). U2AF65 is involved in mechanisms that lead to androgen receptor splicing in prostate cancer cells and regulate the stability of mature mRNA in trypanosomes (Gupta et al., 2013; Liu et al., 2013). Sunghee Cho concluded that U2AF65 possesses a splicing inhibitory function that leads to alternative exon skipping (Cho et al., 2015). Jakubauskienë et al. (2015) present experimental evidence that splicing factor U2AF35 and U2AF65 expression levels in gastrointestinal tract (colon, gastric, and pancreatic) tumors differ compared to that in healthy tissues and cell lines derived from corresponding organs. U2AF65 inhibited HPV16 early polyadenylation and enhanced HPV16 late mRNA splicing, thereby activating HPV16 late gene expression (Nilsson et al., 2018). U2AF65 regulates milk synthesis and bovine mammary epithelial cell growth (Yu et al., 2019).

It is worth noting that most of the research on U2AF65A has focused on mammalian life activities and cancer. However, few studies have focused on U2AF65A in plants. The existence of multiple isoforms of U2AF may be quite general in plants because two genes expressing U2AF65 have been identified in *Arabidopsis* and different isoforms of the U2AF small subunit are expressed in rice (Domon et al., 1998). The *Arabidopsis* splicing factors *AtU2AF65*, *AtU2AF35*, and *AtSF1* shuttle between the nucleus and cytoplasm. These proteins also move rapidly and continuously in the nuclei, and their movements are affected by ATP depletion (Park et al., 2017). U2AF65 of *Arabidopsis* regulates flowering time and the growth of pollen tubes, and the *AtU2AF65A* mutant exhibits late flowering (Park et al., 2019). *AtU2AF65B* is involved in ABA-mediated flowering transition in *Arabidopsis* (Xiong et al., 2019). Although there are many studies on U2AF65A, its specific functions are poorly understood, especially in plants. As such, further studies are required to identify plant U2AF65A proteins and determine how these proteins mediate U2 snRNP, which could also involve many U2AF65A interacting proteins. The current study provides a phylogenetic description of plant U2AF65A and acts as a primer to pursue future functional studies in plant alternative splicing regulation. In the present study, we explored the *U2AF65A* gene family across different plant species and analyzed their phylogenetics, conserved gene structure, protein domains, spatiotemporal gene expression profiles, gene localization, and phenotypes in response to stress. This work offers an overview of the phylogenetics, genomics, and expression of this gene family and elucidates its preliminary functions, building a foundation for the further functional characterization of this gene family in *Viridiplantae*.

## Materials and Methods

### Sequence Identification of Plant *U2AF65A* Genes and Phylogenetic Analysis

The *Arabidopsis thaliana* U2AF65A protein (AT4G36690) sequence was used as a query to carry out a protein BLAST search with an *e*-value cut off of 1e^–10^ (Camacho et al., 2009) against all the available plant genome sequences from Phytozome v12.1.6^1^. As a result, 113 putative U2AF65A sequences from 33 plant species were identified for subsequent analysis.

Protein sequences of the aforementioned plant *U2AF65A* genes were obtained from the Phytozome v12.1.6 annotation and applied for phylogenetic tree construction to infer the clustering patterns and evolutionary relationships. For loci with multiple splicing isoforms, the locus with the longest coding sequence was chosen. Subsequently, a multiple sequence alignment of protein sequences was conducted using Muscle v3.8 with default settings (Edgar, 2004). The phylogenetic relationship among plant *U2AF65A* genes was determined according to the maximum-likelihood (JTT + R5 model) method implemented in PHYML 3.0 (Guindon et al., 2010). The online tool ITOL^2^ was used to visualize and modify the resulting phylogenetic tree (Letunic and Bork, 2019).

### Analysis of Gene Structure, Protein Domain and MEME Motif

Gene structure and protein domain information was obtained from Phytozome v12.1 and the Pfam protein family database. The 10 most conserved motifs were obtained using Multiple Expectation Maximization for Motif Elicitation applied on the MEME^3^ server from the input cDNA sequences of plant U2AF65A (Bailey et al., 2009).

### Promoter Motif Prediction and Protein–Protein Interaction Network

The 1.5-kb promoter sequences of plant *U2AF65A* genes were extracted from the Phytozome database and used for the prediction of *cis*-elements using PlantCARE^4^ (Lescot, 2002). The *Arabidopsis thaliana* U2AF65A protein was input for a STRING^5^ network analysis to identify the top interaction partners.

### Subcellular Localization Prediction

PSORT^6^ was used to predict the subcellular localization of the U2AF65A protein in representative plant species.

The full-length CDS of *OsU2AF65A* was amplified using gene-specific primers (5′-CCTGTTGTTTGGTG TTACTTAAGCTTATGGCGGAGCACGAGGAG-3′ and 5′-TC CTCGCCCTTGCTCACCATGGATCCTCAACCGTCGTATTG CCCTTC-3′) and cloned into the PGreenII vector using the ClonExpress II One Step Cloning Kit (Vazyme). Nipponbare rice seedlings grown at approximately 30°C for 1 week were selected, and their stems were collected to extract protoplasts. Equal volumes of the constructed plasmids and extracted protoplasts were mixed with 40% PEG4000 solution at a ratio of 1:5 (*v/v*), cultured overnight at 30°C, and observed under a laser confocal microscope (Leica TCS SP8).

### Plant Growth and Stress Treatment

The seeds of *Arabidopsis thaliana* wildtype Col-0 and *AtU2AF65A* mutants (T-DNA insertion, 273 and 65A-1) were cultured on 1/2MS medium for vertical and horizontal growth in a climate chamber at 23°C and 28°C (16 h/8 h light/dark cycle), respectively. On the 7th day, part of the wildtype and mutant seedlings were transplanted into soil (vermiculite:nutrient soil = 1:3) at 23°C until they grew to the 6–8 leaf stage and then subjected to high-temperature treatment at 40°C for 4 days. The remaining seedlings were photographed, and their data were recorded. Specifically, hypocotyl length at 28°C was measured. On the 9th day, roots were photographed and their length recorded. Similarly, the fresh and dry weights of 20 plants were measured in triplicate.

Nipponbare seeds were first soaked in carbendazim for 1 day and then in water for another day. Subsequently, the seeds were transferred to a climate chamber (at 24°C under 16 h/8 h light/dark cycle) and exposed to stress until they reached the two real leaf stage (22 days). Hydroponics was used in all four stress treatments. The seedlings were grown at 8°C for the cold treatment and supplied with 20% PEG6000 for drought treatment. The seedlings were grown in the presence of 100 mmol⋅L^–1^ sodium chloride for salinity treatment and 100 μmol⋅L^–1^ cadmium sulfate for heavy metal exposure treatment. The seedlings were sampled at 0, 3, 6, and 12 h under stress for RNA extraction and gene expression analysis using RT-qPCR. The seeds of rice mutant lines (65A-11 and 65A-13) obtained using the CRISPR–Cas9 system and of wildtype Nipponbare were sown in soil and grown in an incubator at 23°C for 3 weeks. Thereafter, the seedlings were subjected to high-temperature treatment at 39°C for 11 days. The survival rate was measured. As the standard of survival, a plant was considered dead when all leaves had curled and wilted.

Total RNA was isolated using TRIzol reagent (Invitrogen) and converted to complementary DNA (cDNA) using a Transcriptor First Strand cDNA Synthesis Kit (Roche) following the manufacturer’s protocol. RT-qPCR was performed using a 7500 Real-time PCR Detection System (Bio-Rad) in conjunction with Fast Start Universal SYBR Green Master Mix (Roche). Forward and reverse primers of *OsU2AF65A* were used to produce a single amplification (5′-TGCTGTTGGCTTAACACCTG-3′ and 5′-AACTCCCTGACTTGAGCTTCTG-3′). *OsACTIN* was used as a reference gene to normalize the data, and the relative expression levels were calculated using the 2^–ΔΔ^ ^CT^ method (Cao et al., 2013). All experiments were repeated at least three times.

One-way analysis of variance (ANOVA) was used to estimate the differences in gene expression and observed phenotypes according to Tukey’s *post hoc* test at the *p* < 0.05 level in SPSS 19.0. GraphPad Prism 9.0.0 was used to draw histograms.

### Homology Modeling and Phosphorylation Site Prediction

Homology modeling was conducted by using the *Arabidopsis thaliana* U2AF65A protein sequence based on template 6xlv.1 (crystal structure of leukemia-associated N196K mutant of U2AF65 bound to AdML splice site) through SWISS-MODEL^7^ (Waterhouse et al., 2018). TIMETREE^8^ was used to build a time phylogenetic tree, and the prediction of protein phosphorylation sites was performed using a specific website^9^ (Guo et al., 2020).

## Results

### Genome-Wide Identification and Phylogenetic Comparison of the Plant *U2AF65A* Gene Family

**FIGURE 1:**
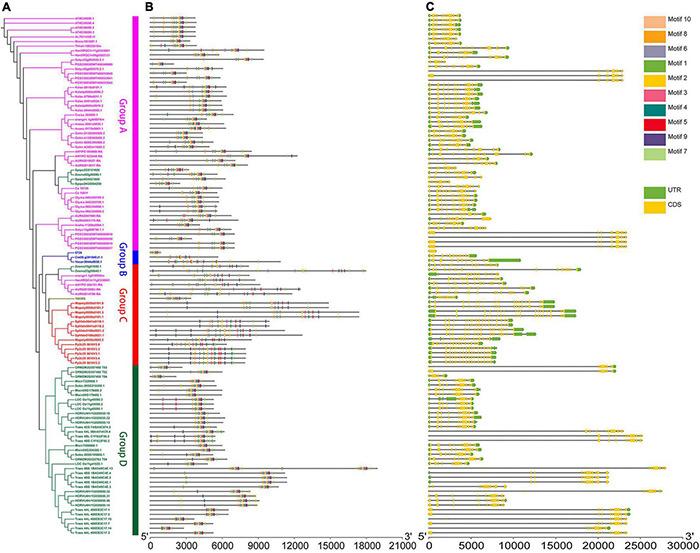
Genomic structure organization and conserved DNA motifs amongst plant *U2AF65A* genes. Gene structure **(A)** and conserved motifs **(B)** of cDNA (by MEME analysis) are shown against the vertical phylogenetic tree **(C)**.

To identify *U2AF65A* genes in different plant species, we carried out a BLAST search using the *Arabidopsis thaliana* U2AF65A protein sequence against the Phytozome database (v12.1.6). After filtering sequences, 113 putative U2AF65A sequences from 33 different plant species were obtained. The sequences were divided into four groups (Figure 1A) and five species, including 18 dicotyledons in pink, 8 monocotyledons in green, 1 fern in brown, 3 algae in blue, and 3 bryophytes in red. For the distribution of the number of genes in these 33 species, one gene was found in nine species, two genes were found in five species (*Anacardium occidentale, Cicer arietinum, Citrus sinensis, Kalanchoe fedtschenkoi*, and *Sorghum bicolor*), three genes were found in five species (*Solanum lycopersicum, Amaranthus hypochondriacus, Helianthus annuus, Spirodela polyrhiza*, and *Zostera marina*), four genes were found in seven species, five genes were found in four species (*Chenopodium quinoa, Miscanthus sinensis, Marchantia polymorpha*, and *Physcomitrella patens*), seven genes were found in one species (*Hordeum vulgare*), eight genes were found in one species (*Solanum tuberosum*), and 15 genes were found in one species (*Triticum aestivum*) (Supplementary Table 1).

The gene copy number and species were mapped to the phylogenetic tree. There were 18 dicotyledons. The pink subfamily contained five species with a single copy of gene, four species with a double copy, three species with three copies, four species with four copies, one species with six copies, and one species with eight copies. In addition, the green subfamily contained eight monocotyledons, including one species with a double copy, two species with three copies, two species with four copies, one species with five copies, one species with seven copies, and one species with 15 species. The blue subfamily contained three algae, which each had a single copy. The brown subfamily contained one fern with a single copy. The red subfamily contained three bryophytes, and one species had four copies and two species had five copies.

Moreover, 113 sequences from 33 plant species may provide us with a more complete phylogenetic relationship of the *U2AF65A* gene family. The red subfamily representing bryophytes and brown subfamily representing ferns are closely related to the blue subfamily representing algae. However, the green subfamily representing monocotyledons is far from the three subfamilies mentioned above and closely related to the pink subfamily representing dicotyledons. Multiple sequences in the same species tend to cluster closely in the same subfamily. Interestingly, in addition to dicotyledons in group A, two monocotyledons (*Spirodela polyrhiza* and *Zostera marina*) also appeared in group A. In addition to bryophytes, group C also contained one fern (*Selaginella moellendorffii*), one monocotyledon (*Zostera marina*), and four dicotyledons (*Chenopodium quinoa*, *Amaranthus hypochondriacus*, *Helianthus annuus*, and *Citrus sinensis*). This interesting phenomenon may indicate the degree of evolution in different species. Except for the example above, subfamilies strictly correspond to the plants they represent.

### Comparative Analysis of Gene Structure and DNA Motifs

Furthermore, to analyze the evolutionary path and identify the conserved functions of *U2AF65A* genes in plants, it is essential to compare their gene structure and conserved gene motifs. To visualize this, the gene structure models and corresponding conserved motifs of the above sequences were attached to the phylogenetic tree (Figure 1). Surprisingly, more than 80 sequences showed a 12 exon–11 intron organization (Figure 1C). Given that the proportion is close to 70%, these sequences appear to be highly conserved in the plant genomic structure across different species, which may suggest strong functional conservation for *U2AF65A* genes. Most of the remaining sequences have 11–15 exons with a few exceptions. For example, *Zostera marina* has 17 exons. It should be noted that there are a few sequences with one intron or no intron. For instance, two sequences of *Triticum aestivum* have one intron while *Selaginella moellendorffii* and four sequences of *Solanum tuberosum* have no introns. In addition, Group A and Group B presented a smaller the gene structure and Group B and Group C were different in size and did not show an obvious trend.

To further study the characteristic regions of the *U2AF65A* genes, their motifs were analyzed using the MEME motif search tool. The results demonstrated that 99% of the sequences (105 sequences) exhibited similar sequence signatures and contained 9–10 of the top 10 identified motifs (Figure 1B and Supplementary Figure 1). The remaining one sequence (*Ostreococcus lucimarinus*) had seven motifs. Thus, the gene structure of *U2AF65A* genes may have some subtle relationship with conserved motifs.

### Protein Domains, Homology Modeling and Phosphorylation Site Prediction

**FIGURE 2:**
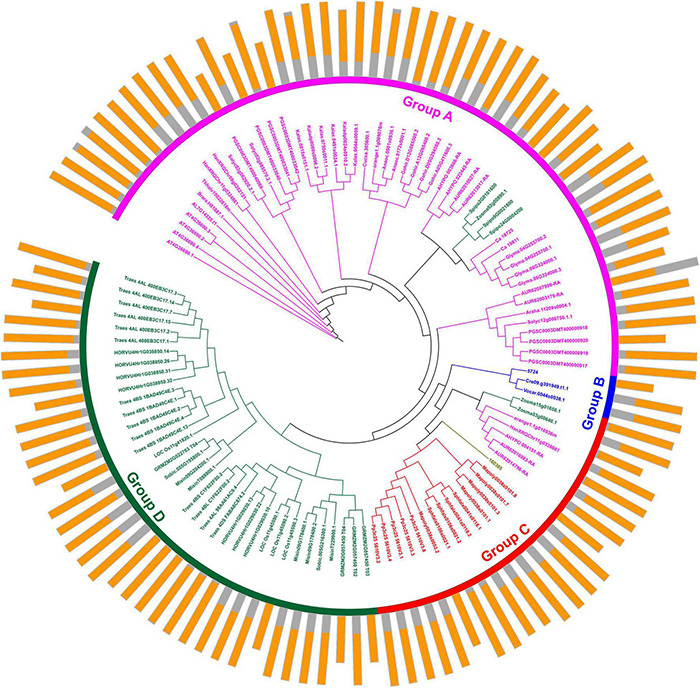
Protein structure organization amongst plant *U2AF65A* genes. The protein structure (outer ring) is displayed on the outermost side, and the amino acid sequence extends from the inside to the outside, the yellow parts of the bar represent the RNA-recognition motif (RRM), and the gray parts of the bar represent the full-length amino acid sequence of the protein. A circular phylogenetic tree (inner ring) is displayed inside.

Protein domains were analyzed to further study the characteristics of the U2AF65A proteins (Figure 2). A total of 113 peptides were predicted to have an N-terminal domain annotated as RRM1 (Figure 2, outer ring). The sequence of all species contained RRM1, which further reflects that the U2AF65A protein has an important role in the normal life activities of plants and may be related to their ability to bind to RNA. The differences in protein domains are very small, which may indicate that the protein has the same function in different species.

**FIGURE 3:**
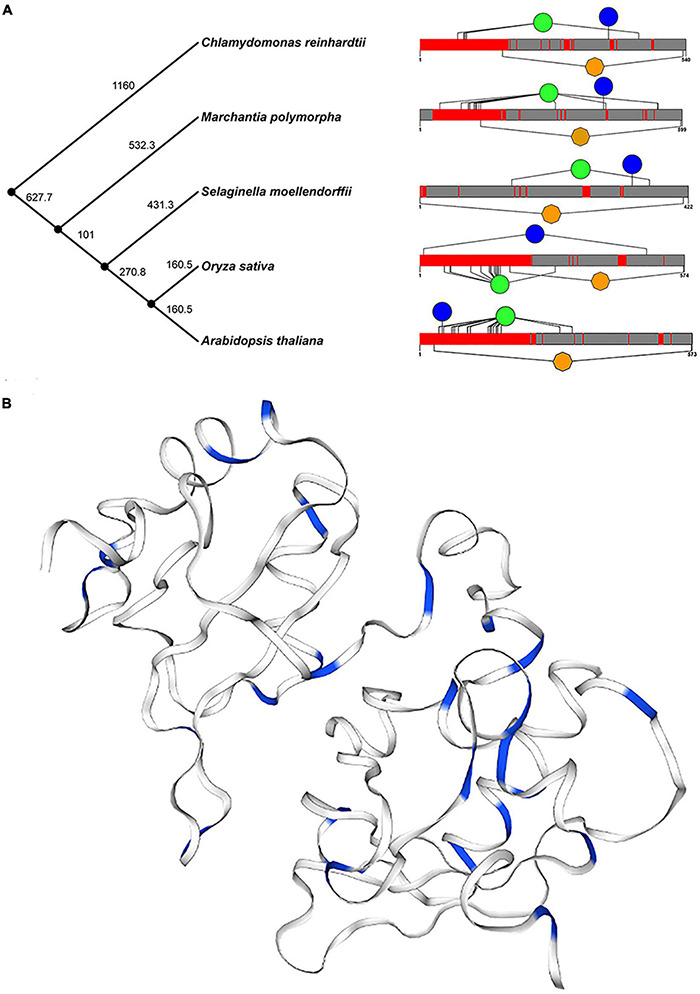
Conservative analysis of plant U2AF65A proteins. **(A)** Protein structures (right panel) in different plants are shown against time on the phylogenetic tree (left panel) of plants. Orange represents the RRM domain, red represents disorder, blue represents Tyr, and green represents Ser. **(B)** The three-dimensional structure of plant U2AF65A protein is generated (using *Arabidopsis* protein sequence) and represented. Blue indicates Ser and Thr in the amino acid sequence.

In addition, we selected five plants (*Arabidopsis thaliana, Oryza sativa, Chlamydomonas reinhardtii, Selaginella moellendorffii*, and *Marchantia polymorpha*) to further determine the structure and function of U2AF65A proteins. The time phylogenetic tree (Figure 3A, left panel) and 2-D map (Figure 3A, right panel) were built. Generally, the U2AF65A protein contains an RRM1 domain in orange and many different lengths of disordered structures in red. We predicted the phosphorylation sites on the website based on the amino acid sequences of the above five species and then selected the sites whose values were greater than or equal to 99% phosphorylation potential value based on the data (Supplementary Figure 2). The results are shown on the 2-D map, with blue representing tyrosine and green representing serine. Interestingly, all five species showed abundant serine, which may be related to phosphorylation, and less tyrosine.

Furthermore, a 3-D model of plant U2AF65A proteins was constructed based on the template by using a homology modeling approach (Figure 3B). In the model, the amino acids related to phosphorylation, serine, and threonine are marked in blue. Both 2-D and 3-D models have the same trend, which fully implies that U2AF65A may function by participating in phosphorylation.

### U2AF65A Subcellular Localization

**FIGURE 4:**
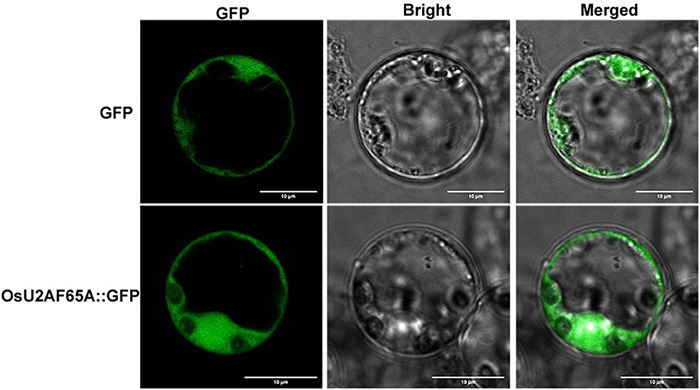
Analysis of the subcellular localization of *OsU2AF65A. OsU2AF65A* is localized to the nucleus and cytoplasm. The plasmid containing *OsU2AF65A*-GFP or GFP was transformed into rice protoplasts. Examination was performed under a confocal laser scanning microscope in the dark field for green fluorescence **(left)**, white field **(middle)** for cell morphology and in combination **(right)**, respectively; bar = 10 μm. Experiments were repeated three times with similar results.

The subcellular localization of *OsU2AF65A* was determined by transiently expressing green fluorescent protein-labeled OsU2AF65A in rice protoplasts. *OsU2AF65A* is expressed in the nucleus and cytoplasm of rice protoplasts, and the fluorescence signal in the nucleus was stronger than that in the cytoplasm (Figure 4). In addition, exploring the cellular localization of gene expression is essential to gain a deeper understanding of protein functions. Accordingly, to further understand the sites of U2AF65A protein function in plant cells, we selected 19 species belonging to five subfamilies for the prediction of protein subcellular localization. The results indicated that U2AF65A proteins are nuclear localization proteins in 14 species. This finding is consistent with our localization results for U2AF65A in rice. However, U2AF65A is a peroxisomal localization protein in *Spirodela polyrhiza* and may be a cytoplasmic or mitochondrial localization protein for *Ostreococcus lucimarinus*, *Volvox carteri*, *Selaginella moellendorffii*, and *Triticum aestivum* (Supplementary Figure 3).

### Promoter Analysis

To further investigate the regulation of plant *U2AF65A* genes at the transcription level, the 1.5-kb promoter sequence of each plant *U2AF65A* gene was analyzed using the online software PlantCARE. A total of 13577 elements were identified, including 1174 blank elements and 1344 unnamed elements, which accounted for 8.6 and 9.9%, respectively. After screening, 2085 (15.4%) elements were removed. Finally, 8974 elements accounting for 66.1% were left for further analysis. We classified them into four categories by function (Supplementary Figure 4): hormone response (926 elements), light response (579 elements), regulation of basic transcription (6323 elements) and stress response (1146 elements). The hormone-responsive elements included 220 ABREs, with 196 as-1, 196 CGTCA motifs, 118 EREs and 196 TGACG motifs. The light-responsive element consists of 200 Box 4, 242 G-boxes and 137 GT1 motifs. The most abundant regulatory elements of basic transcription include three common elements: 3210 TATA boxes, 2729 CAAT boxes and 384 AT-TATA boxes. The rest of the stress-responsive elements were divided into four categories, with 185 AREs, 412 MYBs, 306 MYCs and 243 STREs.

**FIGURE 5:**
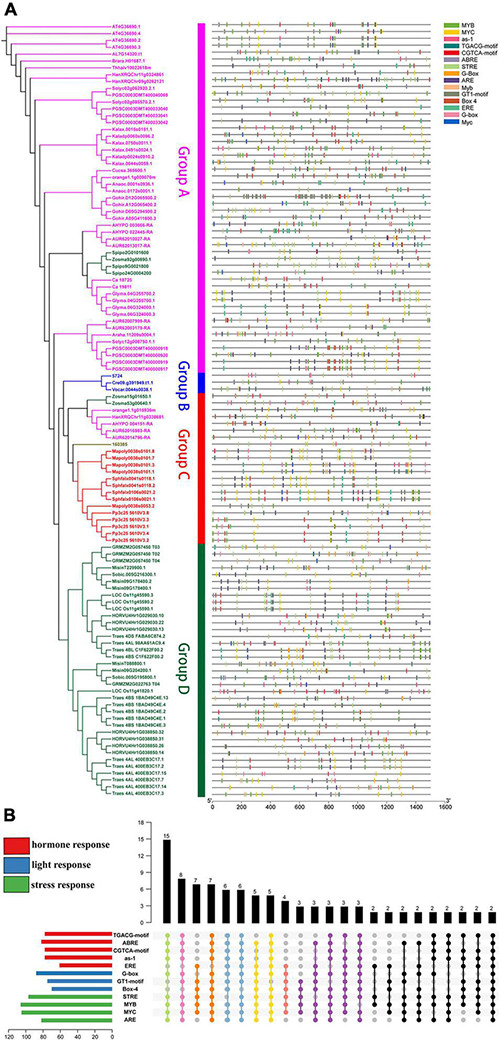
Putative motifs in the promoter regions of plant *U2AF65A* genes. **(A)** Motifs are represented by rectangles of various colors. These *cis*-acting motifs of each plant *U2AF65A* gene are labeled along the 1.5-kb promoter region (straight line) according to their relative nucleotide positions to the transcript start site. **(B)** Specific enrichment statistics of the motifs in response to stress, hormones, and light.

Moreover, the distribution of various elements in the same group is relatively uniform. Group A, Group C and Group D contain abundant stress-related elements (Figure 5A), while Group B (*Chlamydomonas reinhardtii, Ostreococcus lucimarinus*, and *Volvox carteri)* contains few elements. Therefore, Group B can only respond to a small stimulus, which may be related to the evolution of these species. A comprehensive analysis (Figure 5B) suggested that there were only four sequences with all elements at the same time. However, there were 49 sequences with 9–11 elements and 15 sequences with 5–8 elements at the same time. These results indicate that *U2AF65A* genes can respond to a variety of stimuli and have a complex response network.

### Interaction Network

**FIGURE 6:**
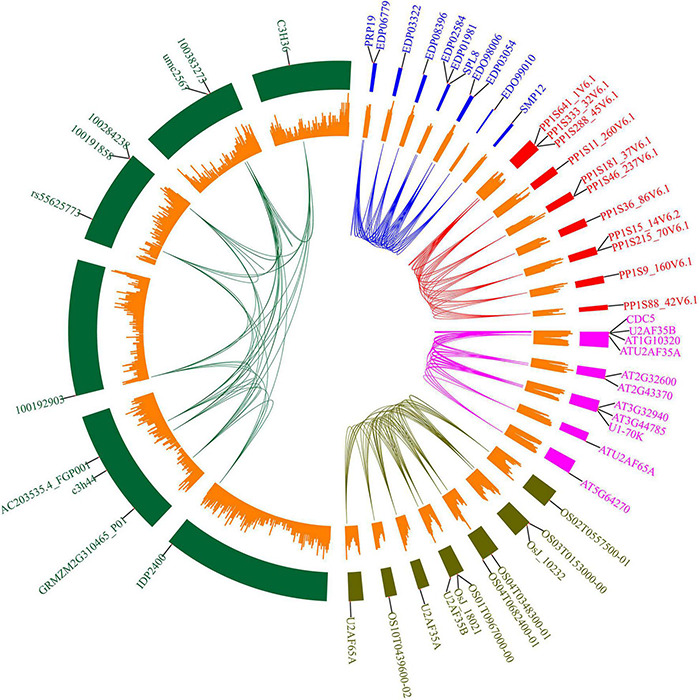
Protein interacting-partners of *Arabidopsis thaliana, Oryza sativa, Zea mays, Physcomitrella patens*, and *Chlamydomonas reinhardtii* U2AF65A proteins. Protein interaction networks within species were connected by lines of corresponding colors. Red represents *Physcomitrella patens*, green represents *Zea mays*, blue represents *Chlamydomonas reinhardtii*, pink represents *Arabidopsis thaliana*, and brown represents *Oryza sativa.* The small orange rectangle represents the number of genes contained in the chromosome region. The outer label represents the genes encoding the interacting proteins.

To further analyze the potential role of U2AF65A proteins, we selected five common species (*Arabidopsis thaliana, Oryza sativa, Zea mays, Physcomitrella patens*, and *Chlamydomonas reinhardtii*) and built an interaction network (Figure 6) based on data obtained from STRING. As a result, we obtained 10 interacting proteins for each species (Supplementary Table 2). The numbers of interacting proteins showed obvious consistency, which may suggest that the U2AF65A protein has a fairly complex interaction network in different plants. Regarding the genes encoding the corresponding proteins, there was an interesting phenomenon in which genes in *Oryza sativa, Chlamydomonas reinhardtii*, and *Physcomitrella patens* were evenly distributed on seven chromosomes, while those in *Arabidopsis thaliana* and *Zea mays* were concentrated on a few chromosomes. Furthermore, among the 10 proteins interacting in *Physcomitrella patens*, there were 2 SNRPD2 (small nuclear ribonucleoprotein D2) proteins and 1 SF1 and 7 proteins with unknown function. However, 10 proteins were related to splicing in *Chlamydomonas reinhardtii*. In the interaction network of *Zea mays* and *Arabidopsis thaliana*, in addition to the proteins related to splicing, some proteins were related to cell division. Interestingly, in the interaction network of *Oryza sativa* and *Arabidopsis thaliana*, zinc finger domain-containing proteins appeared in the network. In terms of the types of interacting proteins, the U2AF65A protein also showed a high degree of consistency in different plants. Furthermore, each plant has a unique interaction protein that is different from other plants, which may be related to the different degrees of evolution and development.

### Expression Patterns of *OsU2AF65A* Under Abiotic Stress

**FIGURE 7:**
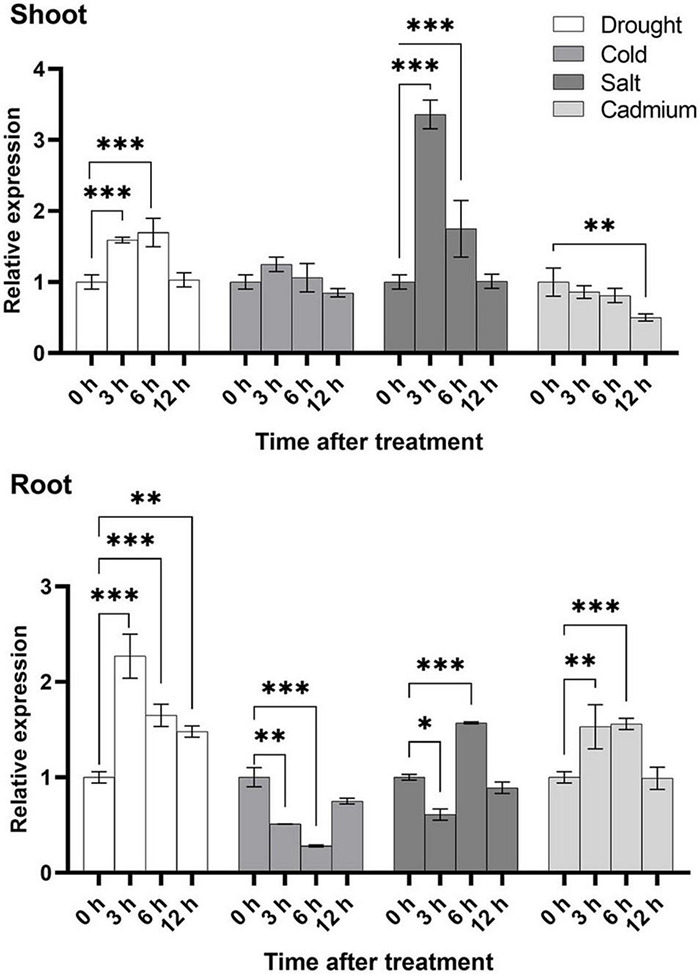
Stress-inducible expression of *OsU2AF65A.* Three-week-old Nipponbare seedlings were exposed to 20% PEG6000, 100 mmol L^–1^ sodium chloride, 100 μmol L^–1^ cadmium sulfate, or cold temperature (8°C). Samples were collected at different time intervals, and the transcript level of *OsU2AF65A* was quantified using RT-qPCR; *LOC_Os11g41820.1* was selected the *U2AF65A* gene in rice. Data are presented as the mean ± SD of three independent experiments and the differences amongst different time points (0, 3, 6, and 12 h) are indicated by asterisks (^∗∗∗^*p* < 0.001, ^∗∗^*p* < 0.01, and ^∗^*p* < 0.05) according to ANOVA and Tukey’s *post hoc* test.

To further study the expression level of *U2AF65A* genes in plants, we selected the four stress treatments based on the results of promoter analysis: cold, drought, salt and Cd^2+^. In general, the expression level in the shoot was higher than that in the root under the cold and salt treatment, while under the drought and cadmium treatment conditions, the expression of the root exceeded that of the shoot (Figure 7), which indicated that *U2AF65A* was specifically expressed in tissues to combat adversity in response to different abiotic stresses. Under the cold treatment, the expression of *U2AF65A* increased slightly at 3 h, decreased to normal levels at 6 h, and then showed no changes in expression. In the roots, the expression decreased to a minimum at 6 h and then restored to a normal level at 12 h. Under the salt treatment, the expression of *U2AF65A* increased significantly and reached a maximum at 3 h, decreased at 6 and 12 h, and returned to normal levels at 12 h in shoots. The expression of *U2AF65A* decreased after 3 h of the salt treatment, increased significantly at 6 h, and then decreased to normal levels at 12 h in roots. The expression of *U2AF65A* increased significantly at 3 and 6 h of drought treatment, reached a maximum at 6 h, but returned to normal levels at 12 h in shoots. The expression of *U2AF65A* increased significantly at 3–12 h of drought treatment and reached a maximum at 3 h in roots. The expression of *U2AF65A* showed a downward trend at 3, 6, and 12 h after the cadmium treatment in shoots. However, it increased significantly at 3 and 6 h after the cadmium treatment, reached a maximum at 6 h and returned to normal levels at 12 h in the roots. In summary, *U2AF65A* was not sensitive to cold in the shoots but was sensitive to salt, drought and cadmium treatment in the shoots and roots. Interestingly, in response to cadmium stress, the shoot and the root showed opposite trends. There may be a complicated regulation network.

### *AtU2AF65A* Mutation Inhibited *Arabidopsis thaliana* Growth

**FIGURE 8:**
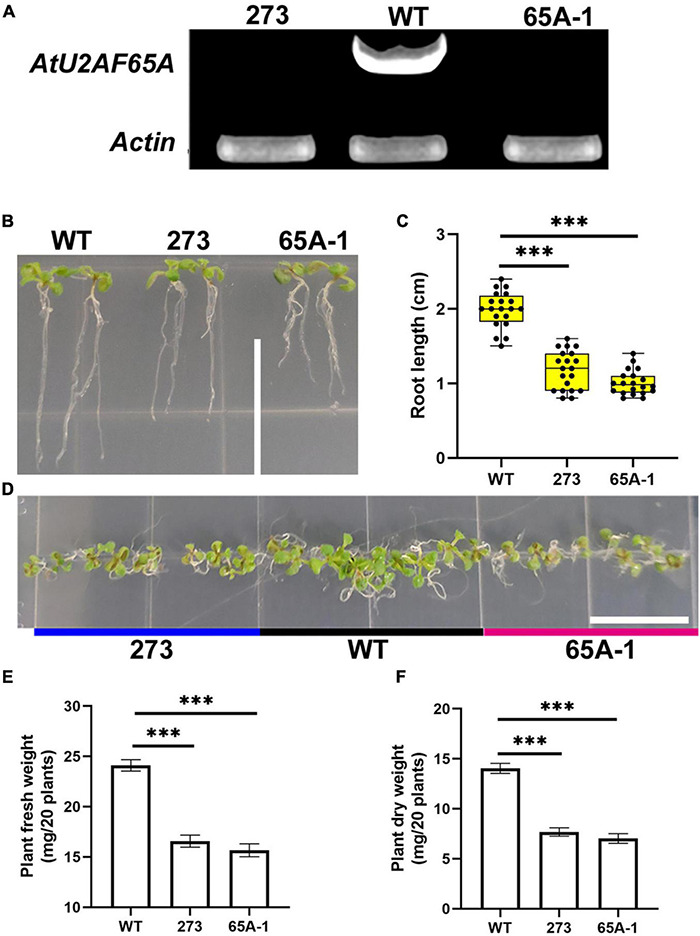
*AtU2AF65A* mutations inhibit plant development. **(A)** *Arabidopsis* mutants were identified using RT-PCR. **(B)** Phenotype of *Arabidopsis thaliana* seedlings growing vertically for 9 days; bar = 14 mm. **(C)** Root length of plants (20 plants per repetition). **(D)** Phenotype of *Arabidopsis thaliana* seedlings growing horizontally for 9 days; bar = 14 mm. **(E)** Fresh weight of 20 *Arabidopsis* plants growing vertically for 9 days. **(F)** Dry weight of 20 *Arabidopsis* plants growing vertically for 9 days. Experiments in **(B,D)** were repeated three times with similar results. Data in **(C,E,F)** are presented as the means ± SD of three independent experiments, and differences between the 273/65A-1 mutants and wildtype are indicated by asterisks (^∗∗∗^*p* < 0.001) according to ANOVA and Tukey’s *post hoc* test.

To further determine the functions of U2AF65A, we purchased *Arabidopsis thaliana* mutants (273 and 65A-1). Four-week-old mutant *Arabidopsis thaliana* plants was sampled and analyzed with RT-PCR using the cDNA template. *U2AF65A* transcripts were not detected (Figure 8A). The roots of the 273 and 65A-1 mutants were significantly shorter than those of the wildtype (Figure 8B). On the 9th day, the root length of the 273 and 65A-1 mutants was decreased by approximately 40% and 48%, respectively (Figure 8C). Similarly, on the 9th day, the *Arabidopsis thaliana* mutants grown horizontally were smaller (Figure 8D). As such, the fresh and dry weight of the 273 mutants was decreased by approximately 30% and 47%, respectively (Figures 8E,F). Similarly, the fresh and dry weight of the 65A-1 mutants was decreased by 33% and 48%, respectively (Figures 8E,F). These results indicate that *AtU2AF65A* plays a regulatory role in *Arabidopsis thaliana* growth.

### *U2AF65A* Mutation Reduced Plant Tolerance of High Temperature in *Arabidopsis thaliana* and Rice

**FIGURE 9:**
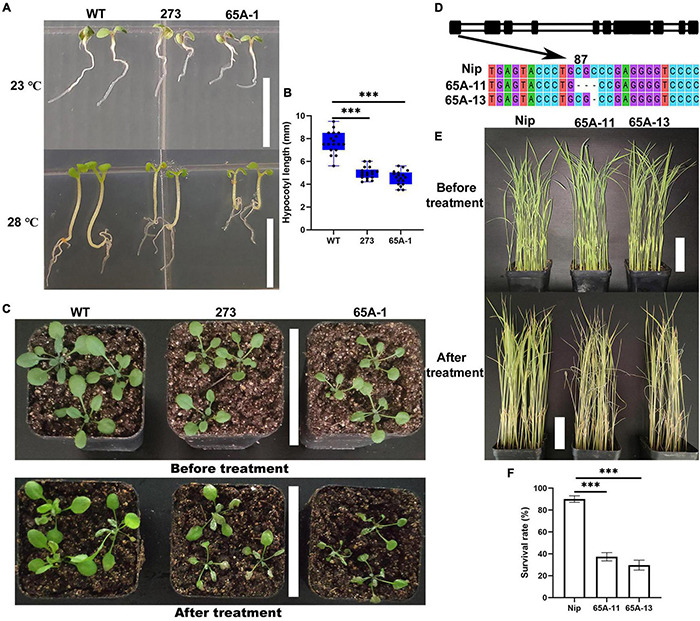
The U2AF65A mutations reduced the tolerance of *Arabidopsis thaliana* and rice plants to high temperature. **(A)** Hypocotyl phenotype of *Arabidopsis thaliana* grown vertically for 7 days at 23°C and 28°C; bar = 10 mm. **(B)** The hypocotyl length of plants (18 plants per replicate). **(C)** Growth of 3-week-old *Arabidopsis* seedlings exposed to high temperature for approximately 4 days; bar = 7 cm. **(D)** Identification of the rice *U2AF65A* mutant. The black column represents the exon sequence of *OsU2AF65A.* The 65A-11 and 65A-13 mutants lost respectively 3 bases (CGC) at position 87 and 1 base **(C)** at position 89 of the first exon. **(E)** Growth of 3-week-old rice plants before and 11 days after the high-temperature treatment; bar = 7 cm. **(F)** Survival rate of rice; plants were curled and withered leaves were considered dead. Experiments **(A,C,E)** were repeated three times with similar results. Data in **(B,F)** are presented as the means ± SD of three independent experiments, and differences between the mutants and wildtype are indicated by asterisks (^∗∗∗^*p* < 0.001) according to ANOVA and Tukey’s *post hoc* test.

*Arabidopsis thaliana* was grown vertically at the normal temperature of 23°C for 7 days, and no differences in the length of the hypocotyl were noted (Figure 9A, upper panel). When the temperature was raised to 28°C on the 7th day, the hypocotyl growth of the 273 and 65A-1 mutants was significantly inhibited compared with that of the wildtype (Figure 9A, lower panel). Specifically, the hypocotyl length of the 273 and 65A-1 mutants was reduced by approximately 36% and 43%, respectively (Figure 9B). Three-week-old *Arabidopsis thaliana* seedlings were exposed to high temperature (40°C) for 4 days. The mutants showed greater leaf curling and wilting than the wildtype (Figure 9C), indicating that the *U2AF65A* mutation reduced the plant tolerance of high temperature. To further determine whether the *U2AF65A* mutation also reduced the tolerance of high temperature in other species, we obtained rice mutants (65A-11 and 65A-13) of the *U2AF65A* gene and screened the homozygous plants through cloning and sequencing. In the 65A-11 mutant, three bases at position 87 of the first exon were lacking, whilst in the 65A-13 mutant, one base at position 89 was lacking (Figure 9D). Subsequently, 3-week-old wildtype and mutant rice plants were exposed to 39°C. After 11 days, the leaves of wildtype seedlings had not curled and wilted, whilst those of most mutants had curled and wilted (Figure 9E). The survival rate of wildtype seedlings exposed to high temperature was as high as 85%, while that of the 65A-11 and 65A-13 mutants was only 37% and 34%, respectively (Figure 9F). These results indicate that rice and *Arabidopsis thaliana* U2AF65A may serve the same regulatory functions in response to high temperature stress, further illustrating that this gene may be functionally conserved.

## Discussion

### U2AF65A Functions Are Highly Conserved

Splicing is a broad process that leads to structural transcript variation and proteome diversity (Chen et al., 2020d; Shen et al., 2021). For a long time, spliceosomes and splicing factors have been considered to be the most promising biological research topics. Many human diseases can be attributed to faulty splicing of genes or regulation of the spliceosome. In recent years, research on splicing in plants has also garnered much interest. For instance, studies on the stress response networks of splicing-related proteins and alternative splicing under abiotic stress have been reported (Chen et al., 2020a, b; Punzo et al., 2020). Splicing plays pivotal roles in plant life (Chen et al., 2020c). As an important cofactor in plants, U2AF65A is involved in the basic development and stress response of *Arabidopsis thaliana*. Moreover, U2AF65A is involved in light-induced germination of *Arabidopsis thaliana* seeds (Tognacca et al., 2019). Here, we confirmed that *AtU2AF65A* is also involved in plant growth following seed germination. The *U2AF65A* mutation impeded plant growth (Figures 8B,D). Interestingly, *AtU2AF65A* is involved in the splicing function of ABA in the regulation of flowering (Wang et al., 2020). Thus, *AtU2AF65A* participates in splicing through the ABA pathway. ABA, a plant inhibitory hormone, promotes seed dormancy. This may explain the involvement of U2AF65A in seed germination following light induction; nonetheless, this warrants further experimental confirmation. Additionally, the alternative splicing of *AtU2AF65A/B* relies upon temperature (Pajoro et al., 2017). The epidermis coordinates the growth of heat-responsive hypocotyls through the PhyB–Pif4–auxin pathway (Kim et al., 2020). Pif4 is a key thermoregulator (Oh et al., 2012), and PhyB is directly associated with the promoters of the key target genes in a temperature-dependent manner (Jung et al., 2016). Our results are consistent with the common regulation of auxin-related gene expression during hypocotyl growth in response to thermal induction. We speculate that U2AF65A is also involved in the PhyB–Pif4–auxin pathway to regulate the early development of *Arabidopsis thaliana.* The splicing induced by U2AF65A differed between the wildtype and phyB mutants, indicating that U2AF65A is involved in splicing through the phyB pathway. Our results proved that U2AF65A is indeed involved in the response of *Arabidopsis thaliana* to high temperature. Under heat induction, the hypocotyl growth of the U2AF65A mutant was inhibited (Figure 9A). Furthermore, we proved that U2AF65A is involved in response to extremely high temperatures. In the present study, the rice and *Arabidopsis thaliana U2AF65A* mutants exhibited reduced tolerance to high temperature (Figures 9C,E). As a splicing cofactor, U2AF65A may affect the splicing of some key genes under heat induction and stress in response to temperature changes. However, the precise molecular mechanisms underlying this process warrant further research. Overall, our findings and previous reports indicate that *AtU2AF65A* is involved throughout the life processes of plants, starting from germination, growth, and development to flowering, in addition to its vital roles in stress response. In addition, we found that the OsU2AF65A protein is localized to the nucleus and cytoplasm and that the AtU2AF65A shuttles between the nucleus and the cytoplasm to perform its functions (Park et al., 2017). These findings indicate that the functions of U2AF65A are conserved. Therefore, U2AF65A regulates the life processes of plants by splicing key genes at different time points. U2AF65A serves crucial functions in plants, which are conserved across different species.

In this work, we successfully identified 113 *U2AF65A* genes from 33 plant species, which ranged from aquatic algae to terrestrial plants. In the phylogenetic tree, plants with a higher degree of evolution and development were closely clustered together, while plants with a lower degree of evolution and development were also closely clustered together. To our surprise, the *U2AF65A* genes in different plants showed consistently high conservation in multiple analyses. Nearly 70% of the sequences showed a 12 exon-11 intron organization, and all peptides were predicted to have an RRM domain. Generally, if a gene is very conserved in many species, then these similarities across species likely indicate that the gene performs some basic functions necessary for many life forms and therefore retains these sequences during evolution. These sequences are generally necessary for life activities, and their mutations often lead to death. Therefore, few mutations are preserved in evolution, and the genes are highly conserved. The same is true for the *U2AF65A* gene, which may perform one or more functions necessary for life activities. Interestingly, the right side of the phylogenetic tree (Figure 1) indicates that UTR sequences exist in many situations. Some genes contain UTR sequences at both ends, some only have UTR sequences at one end, and some do not have UTR sequences at both ends. Notwithstanding, there is no reliable explanation for this phenomenon, which may provide a reference for future research. Our bioinformatic analysis of gene structure and function and protein structure unveiled the involvement of *U2AF65A* in almost all important plant life processes, indicating that this gene is highly conserved in the plant kingdom.

### *U2AF65A* Genes Can Respond to Various External Stimuli

U2AF65A is a splicing cofactor that plays an important role in alternative splicing, and studies have shown that it also plays a role in regulating plant flowering. Few reports have been published to clarify its function in response to abiotic stress. Promoter analysis showed that the complex response elements indicated that U2AF65A could respond to a variety of stimuli, and this result is consistent with the subsequent gene expression analysis. First, a large number of stress-responsive elements were found, including ARE, MYB, MYC, and STRE. As a necessary cis regulatory element of anaerobic induction, AREs play an important role in plants. MYB-MYC is a plant-specific element that is widely involved in the growth, development and response to abiotic stress in plants. It has been reported that STRE is involved in a variety of stress responses in *Neurospora crassa*, including heat, osmotic stress and oxidative stress, and the molecular mechanism mediated by STRE may not be conserved (Freitas et al., 2016). Thus, we designed a corresponding experiment. U2AF65A showed different spatiotemporal expression patterns in rice roots and shoots after different stress treatments, which suggested that there might be some opposite regulatory mechanism of U2AF65A, and it was a relatively long cycle mechanism. In addition to many stress response components, there are a large number of hormone-responsive elements upstream of the *U2AF65A* gene, including ABRE, activating sequence-1, CGTCA motif, ERE and TGACG motif. ABA plays a variety of important roles in plants. In higher plants, (as-1) of the cauliflower mosaic virus 35 S promoter mediates both SA- and IAA-inducible transcriptional activation (Niggeweg et al., 2000). As MJ-responsive elements, CGTCA and TGACG motifs are found in a large number of plants. The estrogen response element (ERE) is a conserved DNA sequence in the promoter of estrogen target genes. It can bind with estrogen receptors, be transcriptionally regulated and is usually located in the promoter of target genes (Govind and Thampan, 2001). These results indicate that the *U2AF65A* gene is widely involved in plant growth, development and stress response. Diverse proteins interact with U2AF65A, including other splicing cofactors and cell division-related proteins. U2AF65A can respond to various external stimuli; however, whether it is involved in protein interactions whilst responding to external stimuli remains unknown. U2AF65A is in amino acids related to protein phosphorylation and may interact with kinases. In the present study, *OsU2AF65A* was involved in the calcineurin B-like protein (CBL)–CBL-interacting protein kinase (CIPK) signaling pathway, which responds to calcium ions (unpublished). Furthermore, our analysis predicting the interacting proteins of U2AF65A identified proteins related to the cell cycle in *Arabidopsis thaliana*, suggesting that U2AF65A regulates the plant cell cycle. Meanwhile, in rice, transport-related proteins were identified as U2AF65A-interacting proteins, indicating that U2AF65A is involved in the related signaling pathways. In plants, ABA signaling is a critical stress responsive pathway (Lin et al., 2020). U2AF65A responds to the ABA pathway during flowering; thus, we speculate that U2AF65A is also involved in the splicing of key genes in the ABA signaling pathway in response to abiotic stresses. Overall, our findings can serve as the reference for elucidating the specific functions of U2AF65A in plants and the molecular mechanisms underlying its functions in abiotic stress.

## Conclusion

In the present study, we identified 113 *U2AF65A* genes from 33 plant species and performed comprehensive bioinformatics analyses of this gene family. The *U2AF65A* genes are highly conserved across different plants and can respond to an array of external stimuli. In the *AtU2AF65A* mutant, the plant size was reduced, root elongation was inhibited, and hypocotyl growth was impaired under heat induction. The OsU2AF65A protein is localized to the nucleus and cytoplasm. In rice and *Arabidopsis*, U2AF65A mutations reduced the plant tolerance of high temperature. Nonetheless, the specific molecular mechanisms involving key genes related to the regulation of splicing for the control of plant growth and stress response warrant further research.

## Data Availability Statement

The original contributions presented in the study are included in the article/Supplementary Material, further inquiries can be directed to the corresponding author/s.

## Author Contributions

SL, CG, YW, YH, and JD performed the material preparation, data collection, and analysis. SL and YC wrote the first draft of the manuscript. YC, BW, HF, HZ, and MC critically revised the manuscript. All authors have contributed to the study conception and design, commented on previous versions of the manuscript, read and approved the manuscript.

## Conflict of Interest

The authors declare that the research was conducted in the absence of any commercial or financial relationships that could be construed as a potential conflict of interest.

## Publisher’s Note

All claims expressed in this article are solely those of the authors and do not necessarily represent those of their affiliated organizations, or those of the publisher, the editors and the reviewers. Any product that may be evaluated in this article, or claim that may be made by its manufacturer, is not guaranteed or endorsed by the publisher.

## Footnotes

1
https://phytozome.jgi.doe.gov/

2
https://itol.embl.de/upload.cgi

3
http://meme-suite.org/tools/meme

4
http://bioinformatics.psb.ugent.be/webtools/plantcare/html/

5
https://string-db.org/

6
https://www.genscript.com/psort.html

7
https://swissmodel.expasy.org/

8
http://www.timetree.org/

9
http://www.cbs.dtu.dk/services/NetPhos/

## Supplementary Material

Supplementary Figure 1 Motifs of the genomic structure. Consensus sequence of the top 10 identified DNA motifs are listed in the ascending order.

Supplementary Figure 2 Prediction of phosphorylation sites in different plants. Values exceeding the threshold indicate the possible involvement in phosphorylation; the higher the value, the greater the probability of involvement in protein phosphorylation.

Supplementary Figure 3 Prediction of the subcellular localization of *U2AF65A* gene expression in different plants. The Protein Subcellular Localization Prediction Tool (PSORT) was used to obtain the probability value of subcellular localization. TBtools was used to draw the heatmap.

Supplementary Figure 4 Classification and statistics of upstream *cis*-acting elements. The *cis*-acting elements retained after the final screening were divided into four categories according to their functions and are displayed in the bar chart.

Supplementary Table 1 Sequence summary of plant *U2AF65A* gene phylogenetic analysis.

Supplementary Table 2 Sequence summary of plant U2AF65A protein-protein interaction network.
