# Supplementary figures and images for: Phylogenetic Analysis of the Plant U2 snRNP Auxiliary Factor Large Subunit A Gene Family in Response to Developmental Cues and Environmental Stimuli
Source: Front Plant Sci. 2021 Nov 17;12:739671. doi: 10.3389/fpls.2021.739671 (PMC8635922; doi:10.3389/fpls.2021.739671)

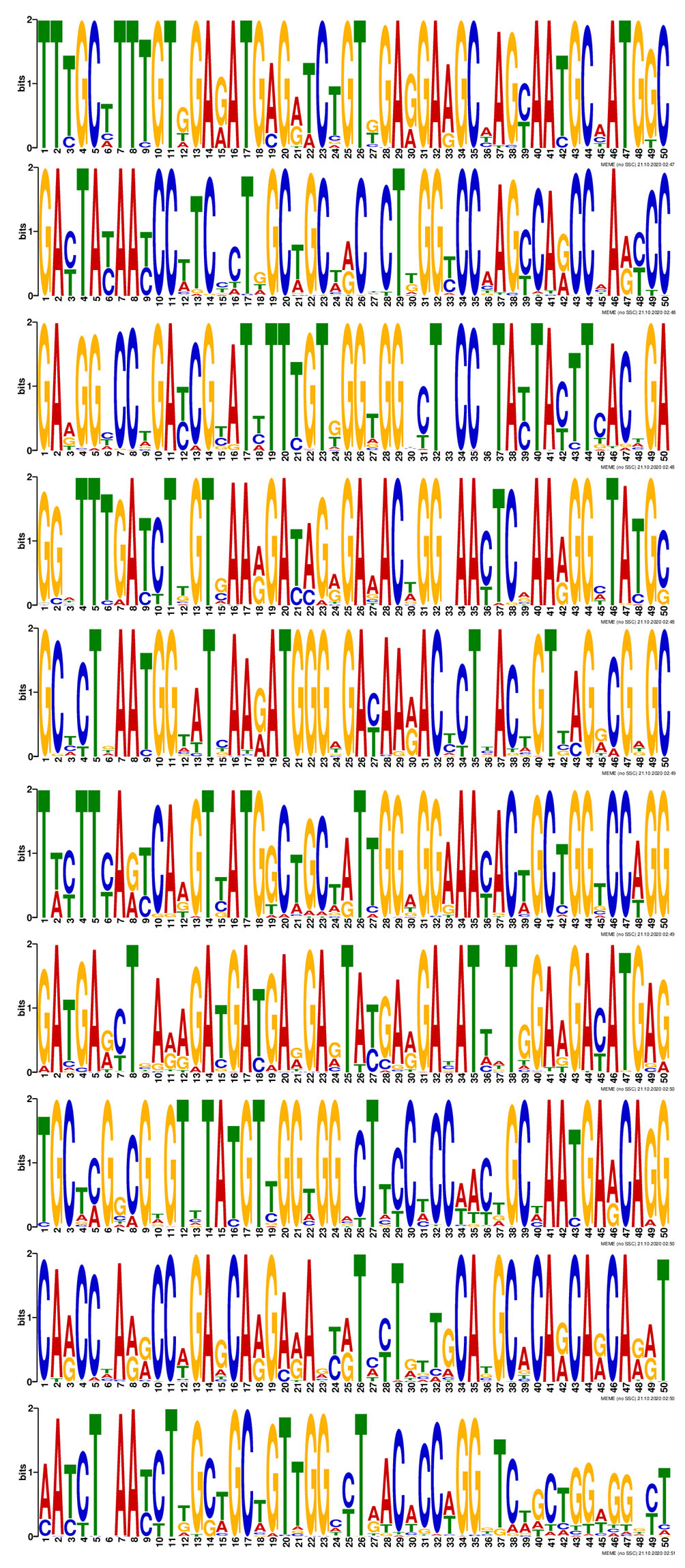

Supplement: Supplementary Figure 1 — Motifs of the genomic structure. Consensus sequence of the top 10 identified DNA motifs are listed in the ascending order. [file Image_1.jpg]

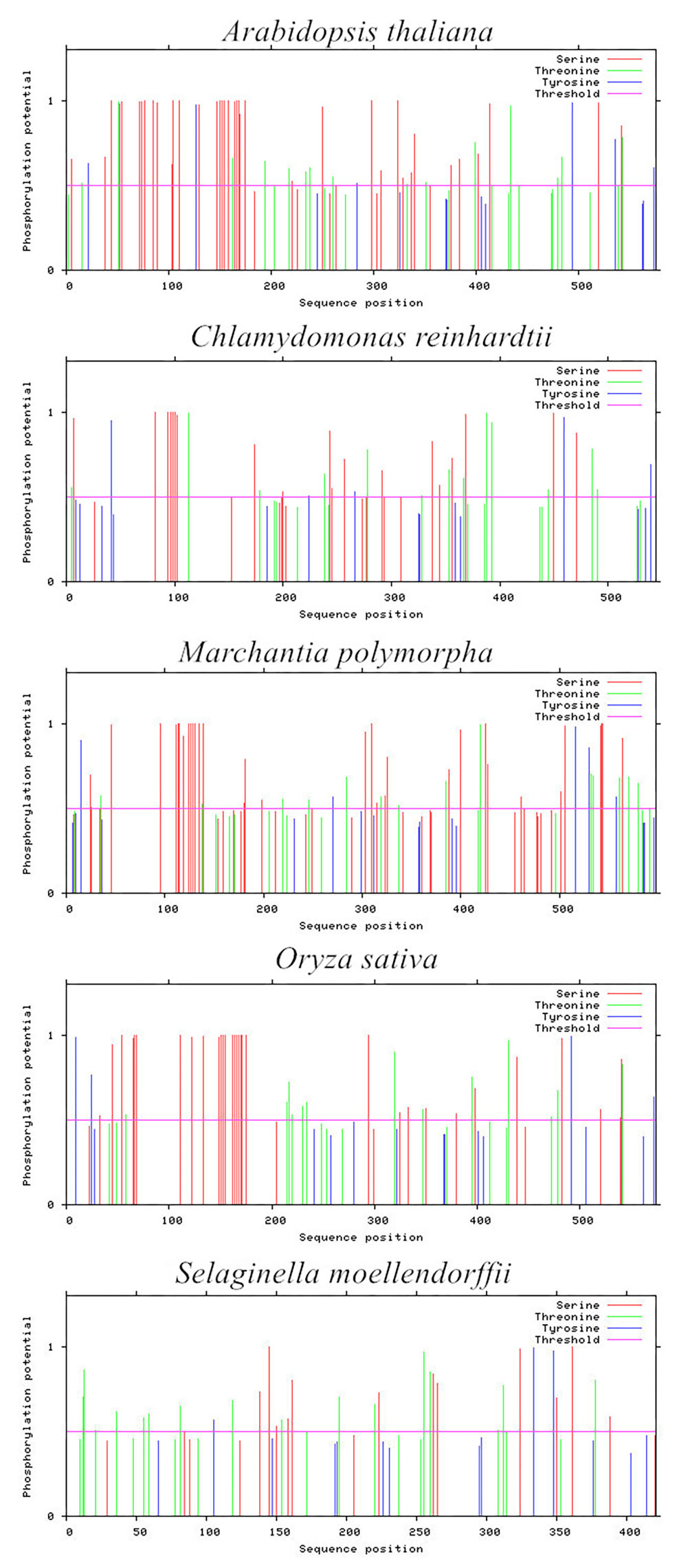

Supplement: Supplementary Figure 2 — Prediction of phosphorylation sites in different plants. Values exceeding the threshold indicate the possible involvement in phosphorylation; the higher the value, the greater the probability of involvement in protein phosphorylation. [file Image_2.jpg]

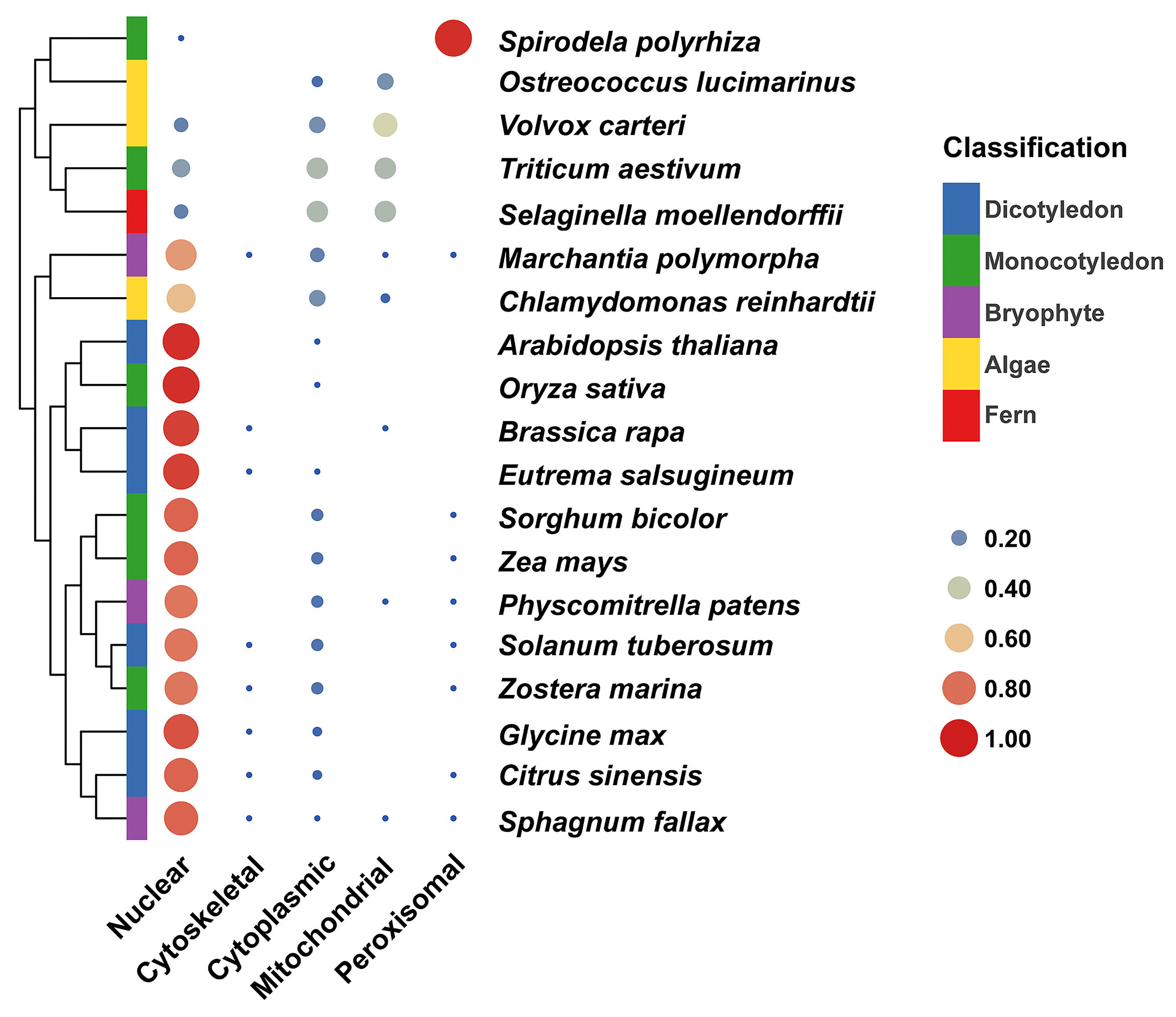

Supplement: Supplementary Figure 3 — Prediction of the subcellular localization of U2AF65A gene expression in different plants. The Protein Subcellular Localization Prediction Tool (PSORT) was used to obtain the probability value of subcellular localization. TBtools was used to draw the heatmap. [file Image_3.jpg]

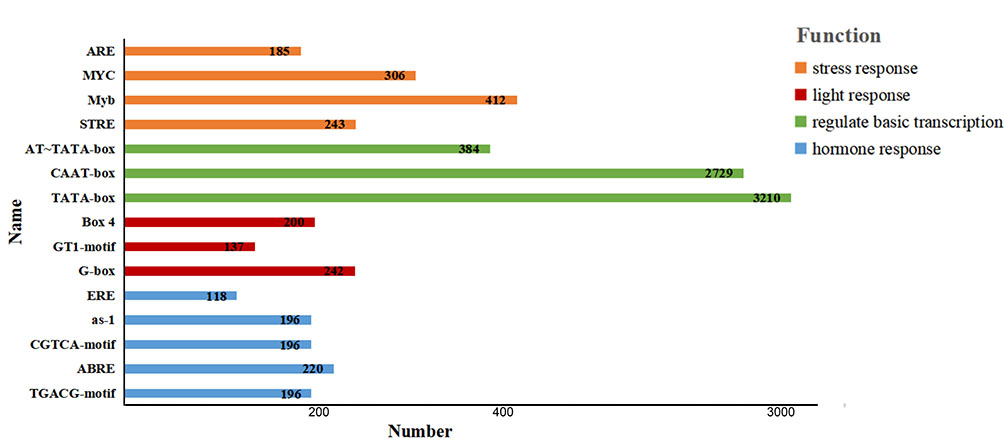

Supplement: Supplementary Figure 4 — Classification and statistics of upstream cis-acting elements. The cis-acting elements retained after the final screening were divided into four categories according to their functions and are displayed in the bar chart. [file Image_4.jpg]
