# Supplementary material for: Phylogenetic Analysis of the Plant U2 snRNP Auxiliary Factor Large Subunit A Gene Family in Response to Developmental Cues and Environmental Stimuli
Source: Front Plant Sci. 2021 Nov 17;12:739671. doi: 10.3389/fpls.2021.739671 (PMC8635922; doi:10.3389/fpls.2021.739671)
Supplement: Supplementary Table 1 — Sequence summary of plant U2AF65A gene phylogenetic analysis. [file Table_1.docx]

**Sequence summary of plant *U2AF65A* gene phylogenetic analysis.**

| **Subfamily** | **Groups** | **Organism** | **Transcript ID** |
| --- | --- | --- | --- |
| **Pink** | **Dicotyledon** | **Solanum lycopersicum** | **Solyc02g085570.2.1** |
|  |  |  | **Solyc02g062920.2.1** |
|  |  |  | **Solyc12g008750.1.1** |
|  |  | **Solanum tuberosum** | **PGSC0003DMT400033041** |
|  |  |  | **PGSC0003DMT400033042** |
|  |  |  | **PGSC0003DMT400033040** |
|  |  |  | **PGSC0003DMT400000918** |
|  |  |  | **PGSC0003DMT400000919** |
|  |  |  | **PGSC0003DMT400000917** |
|  |  |  | **PGSC0003DMT400000920** |
|  |  |  | **PGSC0003DMT400040069** |
|  |  | **Amaranthus hypochondriacus** | **AHYPO_003666-RA** |
|  |  |  | **AHYPO_022445-RA** |
|  |  |  | **AHYPO_004151-RA** |
|  |  | **Anacardium occidentale** | **Anaoc.0001s0936.1** |
|  |  |  | **Anaoc.0172s0001.1** |
|  |  | **Arabidopsis halleri** | **Araha.11209s0004.1** |
|  |  | **Arabidopsis lyrata** | **AL7G14320.t1** |
|  |  | **Arabidopsis thaliana** | **AT4G36690.1** |
|  |  |  | **AT4G36690.2** |
|  |  |  | **AT4G36690.3** |
|  |  |  | **AT4G36690.4** |
|  |  | **Brassica rapa** | **Brara.H01687.1** |
|  |  | **Chenopodium quinoa** | **AUR62010027-RA** |
|  |  |  | **AUR62007999-RA** |
|  |  |  | **AUR62013017-RA** |
|  |  |  | **AUR62003179-RA** |
|  |  |  | **AUR62016983-RA** |
|  |  |  | **AUR62014796-RA** |
|  |  | **Cicer arietinum** | **Ca_18725** |
|  |  |  | **Ca_19811** |
|  |  | **Citrus sinensis** | **orange1.1g009076m** |
|  |  |  | **orange1.1g016936m** |
|  |  | **Cucumis sativus** | **Cucsa.365600.1** |
|  |  | **Eutrema salsugineum** | **Thhalv10022618m** |
|  |  | **Glycine max** | **Glyma.04G255700.2** |
|  |  |  | **Glyma.06G324000.1** |
|  |  |  | **Glyma.04G255700.1** |
|  |  |  | **Glyma.06G324000.3** |
|  |  | **Gossypium hirsutum** | **Gohir.D12G065500.2** |
|  |  |  | **Gohir.A12G065400.2** |
|  |  |  | **Gohir.D05G294500.2** |
|  |  |  | **Gohir.A05G411600.3** |
|  |  | **Helianthus annuus** | **HanXRQChr11g0324861** |
|  |  |  | **HanXRQChr09g0262121** |
|  |  |  | **HanXRQChr11g0338681** |
|  |  | **Kalanchoe fedtschenkoi** | **Kaladp0060s0096.2** |
|  |  |  | **Kaladp0024s0910.2** |
|  |  | **Kalanchoe laxiflora** | **Kalax.0750s0011.1** |
|  |  |  | **Kalax.0015s0151.1** |
|  |  |  | **Kalax.0491s0024.1** |
|  |  |  | **Kalax.0044s0059.1** |
| **Green** | **Monocotyledon** | **Oryza sativa** | **LOC_Os11g41820.1** |
|  |  |  | **LOC_Os11g45590.2** |
|  |  |  | **LOC_Os11g45590.1** |
|  |  |  | **LOC_Os11g45590.3** |
|  |  | **Sorghum bicolor** | **Sobic.005G195800.1** |
|  |  |  | **Sobic.005G216300.1** |
|  |  | **Triticum aestivum** | **Traes_4AL_400EB3C17.14** |
|  |  |  | **Traes_4AL_400EB3C17.3** |
|  |  |  | **Traes_4AL_400EB3C17.1** |
|  |  |  | **Traes_4AL_400EB3C17.2** |
|  |  |  | **Traes_4AL_400EB3C17.7** |
|  |  |  | **Traes_4AL_400EB3C17.15** |
|  |  |  | **Traes_4BS_1BAD49C4E.4** |
|  |  |  | **Traes_4BS_1BAD49C4E.2** |
|  |  |  | **Traes_4BS_1BAD49C4E.1** |
|  |  |  | **Traes_4BS_1BAD49C4E.3** |
|  |  |  | **Traes_4BS_1BAD49C4E.13** |
|  |  |  | **Traes_4DS_FABA6C874.2** |
|  |  |  | **Traes_4BL_C1F622F00.2** |
|  |  |  | **Traes_4BS_C1F622F00.2** |
|  |  |  | **Traes_4AL_98AA61AC9.4** |
|  |  | **Zea mays** | **GRMZM2G022763_T04** |
|  |  |  | **GRMZM2G057450_T03** |
|  |  |  | **GRMZM2G057450_T02** |
|  |  |  | **GRMZM2G057450_T04** |
|  |  | **Hordeum vulgare** | **HORVU4Hr1G038850.26** |
|  |  |  | **HORVU4Hr1G038850.31** |
|  |  |  | **HORVU4Hr1G038850.32** |
|  |  |  | **HORVU4Hr1G029030.22** |
|  |  |  | **HORVU4Hr1G029030.13** |
|  |  |  | **HORVU4Hr1G038850.14** |
|  |  |  | **HORVU4Hr1G029030.10** |
|  |  | **Miscanthus sinensis** | **MisinT229900.1** |
|  |  |  | **MisinT088800.1** |
|  |  |  | **Misin09G204200.1** |
|  |  |  | **Misin09G178400.2** |
|  |  |  | **Misin09G178400.1** |
|  |  | **Spirodela polyrhiza** | **Spipo2G0101600** |
|  |  |  | **Spipo9G0021800** |
|  |  |  | **Spipo24G0004200** |
|  |  | **Zostera marina** | **Zosma53g00640.1** |
|  |  |  | **Zosma92g00890.1** |
|  |  |  | **Zosma15g01650.1** |
| **Blue** | **Algae** | **Chlamydomonas reinhardtii** | **Cre09.g391949.t1.1** |
|  |  | **Ostreococcus lucimarinus** | **5724** |
|  |  | **Volvox carteri** | **Vocar.0044s0038.1** |
| **Brown** | **Fern** | **Selaginella moellendorffii** | **160385** |
| **Red** | **Bryophyte** | **Marchantia polymorpha** | **Mapoly0038s0101.8** |
|  |  |  | **Mapoly0038s0101.3** |
|  |  |  | **Mapoly0038s0101.7** |
|  |  |  | **Mapoly0038s0101.1** |
|  |  |  | **Mapoly0038s0053.2** |
|  |  | **Physcomitrella patens** | **Pp3c25_5610V3.8** |
|  |  |  | **Pp3c25_5610V3.4** |
|  |  |  | **Pp3c25_5610V3.3** |
|  |  |  | **Pp3c25_5610V3.1** |
|  |  |  | **Pp3c25_5610V3.2** |
|  |  | **Sphagnum fallax** | **Sphfalx0041s0118.1** |
|  |  |  | **Sphfalx0041s0118.2** |
|  |  |  | **Sphfalx0106s0021.2** |
|  |  |  | **Sphfalx0106s0021.1** |
