# Supplementary material for: Phylogenetic Analysis of the Plant U2 snRNP Auxiliary Factor Large Subunit A Gene Family in Response to Developmental Cues and Environmental Stimuli
Source: Front Plant Sci. 2021 Nov 17;12:739671. doi: 10.3389/fpls.2021.739671 (PMC8635922; doi:10.3389/fpls.2021.739671)
Supplement: Supplementary Table 2 — Sequence summary of plant U2AF65A protein-protein interaction network. [file Table_2.docx]

**Sequence summary of plant U2AF65A protein-protein interaction network.**

| **Organism** | **Protein Name** | **Description** | **Peptide length** | **Chr** | **Start position** | **End position** |
| --- | --- | --- | --- | --- | --- | --- |
| **Arabidopsis thaliana** | **ATU2AF65A** | **U2 snRNP auxilliary factor, large subunit, splicing factor** | **573** | **4** | **17293992** | **17297722** |
|  | **U2AF35B** | **Zinc finger C-x8-C-x5-C-x3-H type family protein** | **283** | **1** | **3384164** | **3388373** |
|  | **ATU2AF35A** | **U2 auxiliary factor small subunit** | **296** | **1** | **9614551** | **9616357** |
|  | **AT3G44785** | **Zinc finger C-x8-C-x5-C-x3-H type family protein** | **75** | **3** | **16328221** | **16328448** |
|  | **AT5G64270** | **Splicing factor, putative** | **1269** | **5** | **25706659** | **25710925** |
|  | **U1-70K** | **U1 small nuclear ribonucleoprotein 70 kDa** | **427** | **3** | **18826300** | **18829618** |
|  | **AT2G43370** | **U11/U12 small nuclear ribonucleoprotein 35 kDa protein** | **333** | **2** | **18013370** | **18015454** |
|  | **CDC5** | **Cell division cycle 5-like protein** | **844** | **1** | **3161841** | **3165360** |
|  | **AT3G32940** | **RNA-binding KH domain-containing protein** | **607** | **3** | **13490945** | **13493787** |
|  | **AT2G32600** | **Hydroxyproline-rich glycoprotein family protein** | **277** | **2** | **13833545** | **13835663** |
|  | **AT1G10320** | **Zinc finger C-x8-C-x5-C-x3-H type family protein** | **757** | **1** | **3384164** | **3388373** |
| **Oryza sativa** | **U2AF65A** | **Splicing factor U2af large subunit A** | **574** | **11** | **25148703** | **25153456** |
|  | **U2AF35B** | **Splicing factor U2af small subunit B** | **304** | **5** | **28080214** | **28082963** |
|  | **U2AF35A** | **Splicing factor U2af small subunit A** | **290** | **9** | **18985507** | **18987209** |
|  | **OS04T0682400-01** | **Os04g0682400 protein** | **1008** | **4** | **34832040** | **34837343** |
|  | **OS02T0557500-01** | **Zinc finger CCCH domain-containing protein 16** | **411** | **2** | **21110728** | **21116484** |
|  | **OsJ_10232** | **Os03g0263500 protein; Splicing factor 3A subunit 2, putative, expressed** | **321** | **3** | **8660124** | **8663021** |
|  | **OS04T0348300-01** | **Os04g0348300 protein** | **972** | **4** | **16579869** | **16587180** |
|  | **OS03T0153000-00** | **transposon protein, putative, CACTA, En/Spm sub-class, expressed** | **78** | **3** | **2922838** | **2931553** |
|  | **OS10T0439600-02** | **Splicing factor, CC1-like family protein, expressed** | **339** | **10** | **15782080** | **15788016** |
|  | **OS01T0967000-00** | **annotation not available** | **103** | **5** | **14449503** | **14452183** |
|  | **OsJ_18021** | **cDNA, clone: J065062N18, full insert sequence** | **105** | **5** | **14449503** | **14452183** |
| **Zea mays** | **100192903** | **U2 snRNP auxiliary factor large subunit** | **539** | **4** | **2116635** | **2122723** |
|  | **100191858** | **Splicing factor U2af subunit isoform 1** | **307** | **6** | **164773159** | **164775972** |
|  | **100284238** | **Splicing factor U2af 38 kDa subunit** | **299** | **6** | **164773159** | **164775972** |
|  | **C3H36** | **Splicing factor U2af subunit isoform 1** | **304** | **8** | **69658649** | **69660585** |
|  | **c3h44** | **annotation not available** | **172** | **3** | **130398102** | **130399300** |
|  | **umc2567** | **Splicing factor U2af small subunit A** | **287** | **7** | **133562993** | **133564875** |
|  | **GRMZM2G310465_P01** | **annotation not available** | **514** | **3** | **46839500** | **46843662** |
|  | **AC203535.4_FGP001** | **CDC5 protein** | **925** | **3** | **157551793** | **157560623** |
|  | **100383273** | **U11/U12 small nuclear ribonucleoprotein 35 kDa protein** | **483** | **7** | **139032260** | **139036156** |
|  | **IDP2400** | **Inactive protein FRIGIDA** | **284** | **1** | **298511319** | **298515696** |
|  | **rs55625773** | **Small nuclear ribonucleoprotein Sm D2** | **116** | **6** | **38268738** | **38270759** |
| **Chlamydomonas reinhardtii** | **SPL8** | **U2 snRNP auxiliary factor large subunit** | **306** | **9** | **3858613** | **3864236** |
|  | **EDP08396** | **U2 snRNP auxiliary factor, small subunit** | **273** | **6** | **286106** | **288129** |
|  | **EDP03054** | **U2 snRNP auxiliary factor, large subunit** | **446** | **12** | **8374224** | **8378061** |
|  | **EDP03322** | **pre-mRNA-splicing factor CDC5/CEF1 (CDC5L, CDC5, CEF1)** | **811** | **3** | **6672364** | **6677025** |
|  | **EDO98006** | **Nuclear pre-mRNA splicing factor, component of splicing factor 3b** | **1085** | **12** | **377413** | **390437** |
|  | **PRP19** | **Spliceosome component, nuclear pre-mRNA splicing factor** | **503** | **2** | **72872** | **76205** |
|  | **EDP06779** | **U2 snRNP auxiliary factor, large subunit** | **309** | **2** | **2759486** | **2769767** |
|  | **EDO99010** | **PTHR23139 - RNA-BINDING PROTEIN** | **80** | **13** | **5179943** | **5185979** |
|  | **SMP12** | **U1 small nuclear ribonucleoprotein** | **394** | **16** | **1937083** | **1940692** |
|  | **EDP02584** | **splicing factor 1 (SF1)** | **259** | **9** | **131006** | **138736** |
|  | **EDP01981** | **splicing factor 1 (SF1)** | **110** | **9** | **131006** | **138736** |
| **Physcomitrella patens** | **PP1S88_42V6.1** | **annotation not available (573 aa)** | **573** | **25** | **3643498** | **3649839** |
|  | **PP1S36_86V6.1** | **annotation not available** | **768** | **14** | **8619675** | **8627043** |
|  | **PP1S641_1V6.1** | **Predicted protein** | **807** | **3** | **12218406** | **12224144** |
|  | **PP1S9_160V6.1** | **K13095 - splicing factor 1 (SF1)** | **462** | **20** | **3746950** | **3750754** |
|  | **PP1S181_37V6.1** | **small nuclear ribonucleoprotein D2 (SNRPD2, SMD2)** | **108** | **12** | **11589515** | **11591919** |
|  | **PP1S288_45V6.1** | **Predicted protein** | **108** | **3** | **17799381** | **17801683** |
|  | **PP1S46_237V6.1** | **small nuclear ribonucleoprotein D2 (SNRPD2, SMD2)** | **108** | **12** | **11589515** | **11591919** |
|  | **PP1S15_14V6.2** | **Predicted protein** | **327** | **16** | **9538700** | **9541308** |
|  | **PP1S215_70V6.1** | **Predicted protein** | **327** | **16** | **9538700** | **9541308** |
|  | **PP1S333_32V6.1** | **annotation not available** | **803** | **3** | **17285131** | **17290802** |
|  | **PP1S11_260V6.1** | **Predicted protein** | **1292** | **11** | **9589444** | **9594254** |
